# Comparing fourteen consensus biomarkers of aging: epigenetic pace of aging as the strongest predictor of mortality in BASE-II

**DOI:** 10.1186/s40364-026-00909-z

**Published:** 2026-03-06

**Authors:** Valentin Max Vetter, Marit Philine Junge, Christian A. Drevon, Thomas E. Gundersen, Jan Homann, Christina M. Lill, Ulman Lindenberger, Graham Pawelec, Lars Bertram, Denis Gerstorf, Ilja Demuth

**Affiliations:** 1 Charité – Universitätsmedizin Berlin, corporate member of Freie Universität Berlin and Humboldt-Universität zu Berlin, Department of Endocrinology and Metabolic Diseases (including Division of Lipid Metabolism), Biology of Aging working group, Augustenburger Platz 1, 13353 Berlin, Germany; 2 Lübeck Interdisciplinary Platform for Genome Analytics (LIGA), University of Lübeck, Lübeck, Germany; 3 Vitas Ltd. Oslo Science Park, Oslo, Norway; 4 Department of Nutrition, Institute of Basic Medical Sciences, Faculty of Medicine, University of Oslo, Oslo, Norway; 5 Institute of Epidemiology and Social Medicine, University of Münster, Münster, Germany; 6 Ageing Epidemiology Research Unit, School of Public Health, Imperial College, London, UK; 7 Center for Lifespan Psychology, Max Planck Institute for Human Development, Berlin, Germany; 8 Max Planck UCL Centre for Computational Psychiatry and Ageing Research, Berlin, Germany; 9 Institute of Immunology, University of Tübingen, Tübingen, Germany; 10 Health Sciences North Research Institute, Sudbury, ON Canada; 11 Department of Psychology, Humboldt University Berlin, Berlin, Germany; 12 Charité - Universitätsmedizin Berlin, BCRT - Berlin Institute of Health Center for Regenerative Therapies, Berlin, Germany

**Keywords:** Biomarkers of aging, Mortality, Longevity, Cox regression, Epigenetic clock, DunedinPACE

## Abstract

**Background:**

In many countries, lifespan has been increasing faster than healthspan, leading to more years spent with late-life disease and highlighting the need for reliable biomarkers to measure biological aging.

**Methods:**

We used data from the Berlin Aging Study II (BASE-II, 60–80 years of age at baseline, average follow-up 7.4 ± 1.5 years, range 3.9–10.4, *n* = 1,083) to compare 14 biomarkers of aging recently consented by an expert panel for the use as outcome measures in intervention studies: *physiological* (insulin-like growth factor 1 (IGF-1), growth-differentiating factor-15 (DNA methylation derived, DNAmGDF15)), *inflammatory* (high sensitivity C-reactive protein (CRP), interleukin-6 (IL-6)), *functional* (muscle mass, muscle strength, hand grip strength (HGS), Timed-Up-and-Go (TUG), gait speed, standing balance test, frailty phenotype (FP), cognitive health, blood pressure), and *epigenetic* (epigenetic clock, DunedinPACE). Cox proportional hazard regression analyses were performed to investigate their role in prediction of all-cause as well as cause-specific mortality. Results were adjusted for age, sex, lifestyle factors, and genetic ancestry.

**Results:**

In adjusted models of all-cause mortality, HGS, IL-6, standing balance, cognitive health, and the epigenetic clock (DunedinPACE) statistically significantly predicted mortality, with the epigenetic clock (DunedinPACE) emerging as the strongest predictor. CRP, gait speed, IGF-1, blood pressure, muscle mass, DNAmGDF15, FP and TUG were not associated with mortality in this study. These results were corroborated in subgroup analyses stratified by cause of death. Feature selection identified a minimal biomarker set consisting of muscle mass, standing balance, and epigenetic clock (DunedinPACE) that predicted mortality with nearly the same discriminative accuracy (C-index = 0.63) as the full model including all biomarkers (C-index = 0.65).

**Conclusion:**

Among the fourteen investigated biomarkers of aging, DunedinPACE emerged with the strongest and most consistent association with mortality.

**Supplementary Information:**

The online version contains supplementary material available at 10.1186/s40364-026-00909-z.

## Introduction

The continuous increase in lifespan in most industrialized countries over the past few decades has led to a demographic shift towards an aging population [1]. Although everyone ages chronologically at the same pace, biological aging occurs at different rates. Whereas some people are affected by age-related decline at an early age, others maintain good health and overall function well into old age.

To assess these differences in biological aging objectively, a variety of biomarkers has been developed. One proposed application of these biomarkers is the detection of increased mortality risk and/or unfavorable health conditions as early as possible. This would allow an early and targeted intervention so that adverse outcomes can be delayed or prevented before they occur rather than treated after clinical manifestation (e.g. reviewed in [2]). Moreover, biomarkers can be used to investigate the effectiveness of interventions (e.g. reviewed in [3]) with shorter follow-up so their effects can be discerned many years before the impact on a clinical phenotype or risk of death. However, due to lacking consensus on the definition of biological aging [4] and the large number of candidate biomarkers available, it remains unclear which biomarkers are best suited for this task [2]. Thus, systematic biomarker validation was identified as a key priority [2].

Accordingly, Perri and colleagues carried out a three-round Delphi procedure in an effort to reach a consensus on biomarkers of aging best suited as outcome measures for intervention studies, resulting in “An Expert Consensus Statement on Biomarkers of Aging for Use in Intervention Studies” in 2025 [5]. In the first round, experts suggested biomarkers suitable for use in intervention studies. In the following two rounds of the Delphi process, a consensus emerged among the participating experts on the following 14 biomarkers: *physiological* (Insulin-like growth factor 1 (IGF-1), growth-differentiating factor-15 (DNA methylation derived, DNAmGDF15)), *inflammatory* (high sensitivity C-reactive protein (CRP), interleukin-6 (IL 6)), *functional* (muscle mass, muscle strength, hand grip strength (HGS), Timed-Up-and-Go (TUG), gait speed, standing balance test, Frailty Phenotype (FP), cognitive health, blood pressure), and *epigenetic* (epigenetic clock). Most of these biomarkers have been known for a long time and/or have been studied extensively in association with health-related outcomes [6–9]. Furthermore, all biomarkers have been described to be associated with mortality (see a non-exhaustive overview of respective publications in Supplementary Table 1). However, due to differences in the study populations, comparability between biomarkers can be limited and, to date, a direct comparison of these 14 biomarkers in the same cohort does not exist.

As noted by Perri et al. “expert consensus identified 14 potential biomarkers of aging, which may be used as outcome measures in intervention studies. Future aging research should identify which combination of these biomarkers has the greatest utility” [5]. To address this research gap, we first examined these 14 biomarkers for their association with mortality among 1,671 participants from the Berlin Aging Study II (BASE-II), a longitudinally followed cohort, allowing direct comparison of effect sizes within the same individuals and addressing a key criterion for biomarkers of aging as defined by the American Federation for Aging Research [10]. Second, subgroup analyses stratified by cause of death are provided to inform future intervention studies in selecting appropriate endpoints for reducing cause-specific mortality. Third, we identified the minimal biomarker combination exhibiting the highest variance explanation to predict mortality in our data set. This is the first study that analyses all 14 consensus-based biomarkers in one study sample.

## Methods

### Study population

The observational longitudinal BASE-II (between 60 and 80 years at baseline) is an interdisciplinary study investigating healthy vs. unhealthy aging trajectories [11]. For the BASE-II baseline assessment, participants were recruited through the Max Planck Institute for Human Development’s participant pool in Berlin, which was originally established through advertisements in local newspapers and public transportation networks. Between 2009 and 2014, the medical baseline assessment (T0) was completed for 1,671 participants in the older age group [11]. On average 7.4 years later (SD: 1.5 years, range: 3.9 to 10.4 years), 1,083 older participants were followed up as part of the GendAge study [12].

All participants gave written informed consent. All assessments at baseline and follow-up were conducted in accordance with the Declaration of Helsinki and approved by the Ethics Committee of the Charité-Universitätsmedizin Berlin (approval numbers EA2/029/09 and EA2/144/16) and registered in the German Clinical Trials Registry as DRKS00009277 (BASE-II) and DRKS00016157 (GendAge). This manuscript was created in accordance with the STROBE guidelines [13].

### Biomarkers

Biomarkers investigated in this study were chosen based on the consensus statement published by Perri and colleagues in 2025 [5]. An overview of all investigated biomarkers and respective methods stratified by timepoints and use in the main as well as the sensitivity analyses are given in Supplementary Table 2. The following biomarkers were investigated:

#### IGF-1

Blood was collected on filter papers (Dried Blood Spot) and IGF-1 was measured by enzyme-linked immunosorbent assay (ELISA) by VITAS Analytical Services, Ltd. Oslo, Norway using AM-248 - Quantitation of IGF-1 in dried blood spots (DBS) using ELISA. In short, punches from DBS containing human whole blood were eluted in phosphate-buffered saline (PBS) at 4 °C overnight. After equilibration to room temperature, the eluates were analyzed using the DG100B Quantikine Human IGF-1 ELISA kit (R&D Systems, Minneapolis, USA) according to the manufacturer’s instructions.

#### CRP

Due to limited data availability, “normal” (non-high sensitivity) CRP was used in our main analyses. It was measured from serum samples using immunoturbidimetry in an accredited standard hospital laboratory (Labor Berlin - Charité Vivantes GmbH, Berlin, Germany) with the Roche/Hitachi cobas c system (COBAS integra, Roche Diagnostics, Indianapolis, USA). hsCRP, obtained by collection of blood spotted on filters (DBS) from VITAS Analytical Services, was measured by Vitas analytical services using AM-438 -Quantification of hsCRP in DBS, Mitra sticks, whole blood and plasma using MSD Mesoscale ECL. In short, one 3.1 mm punch from dried human whole blood samples (DBS) were eluted in 60 µL kit diluent and left standing at 4 °C over night. After bringing the eluate to room temperature, 10 µL of the eluate were diluted in 500 µL kit diluent and analysis was then performed on a MESO^®^ QuickPlex SQ 120 Multiplex Imager using the V-PLEX Human CRP kit (K151STD-2) from MSD, Rockville, Maryland, USA as described in the kit manual.

#### IL-6

IL-6 at baseline was measured using the highly sensitive Cytometric Bead Array flex kit (BD biosciences, San Jose, US) following the manufacturer´s instruction with the addition of a further dilution of the standard measured three times to improve accuracy of the standard curves. Tracking beads and cytometer setup were monitored over time to guarantee constant performance of the flow cytometer (BD LSR-II). IL-6 at T1 was available but measured using a different method (DBS and the MSD U-PLEX platform), and a large fraction of samples (62%) were below the lower limit of quantification. Therefore, this value was not investigated in this study’s sensitivity analyses.

#### Muscle mass

Appendicular lean mass (ALM) was measured with dual-energy X-ray absorptiometry (Hologic^®^ QDR^®^ Discovery™; Hologic, Inc., Bedford, MA, USA) by a trained technician and calculated using the system software (APEX version 3.0.1., Hologic Inc. Bedford, MA, USA). To increase comparability of results between participants, ALM was BMI-standardized. Further information on ALM in BASE-II can be found elsewhere [14].

#### Muscle strength

was calculated as a binary variable from continuously measured HGS using the age- and BMI-specific cut-off values defined by Fried and colleagues as part of their frailty phenotype [15].

#### TUG

Participants were instructed to rise from a standard chair, walk to a line marked three meters away, turn around, return to the chair, and sit down again. The time the participants needed to perform this task was measured in seconds using a stopwatch. Impairment in TUG was considered if the participants exceeded the pre-defined cut-off of 10 s [7].

#### Gait speed

was measured as the time (in seconds) required to walk 4 m on level ground. Thus, higher values reflected slower gait speed.

#### Standing balance test

The Tinetti Mobility Test [16] is a clinical assessment designed to evaluate fall risk in older individuals, with two parts: balance (part 1) and gait (part 2). For this study, only part 1 of the Tinetti Mobility Test was used. It involves tasks such as unsupported standing, turning, and maintaining stability when lightly pushed. Depending on the task, between 0 and 4 points were awarded and summed. A test value below 15 was used to define impairment.

#### Fried’s frailty phenotype

was assessed using the five criteria defined by Fried and colleagues: weight loss, exhaustion, weakness, slow walking speed, and low physical activity [15]. A point was given if the participant provided values in one of the five categories below or above a specific cut-off resulting in a point range between 0 and 5. To implement this index in BASE-II, some small adjustments were made which are described in detail elsewhere [17]. Pre-frail and frail participants (> 0 points) were considered frail for the purpose of this study which is in accordance with a procedure described in BASE-II before [17, 18].

#### Cognitive health

was assessed using the Digit Symbol Substitution Test (DSST), which measures concentration, working speed, visuomotor coordination, and visual short-term memory. Participants are presented with a coding key pairing numbers with unique symbols and are asked to transcribe the corresponding symbol for each number as quickly as possible within 90 s. The total number of correct allocations is recorded as the test score, with higher scores indicating better cognitive performance.

#### Blood pressure

was measured on both arms using an electronic sphygmomanometer (boso-medicus memory; Jung, Willingen, Germany). The mean systolic blood pressure from both arms was calculated and used for analyses. If assessment of blood pressure was possible/available on only one arm, this value was used.

#### Epigenetic clocks

EDTA-added whole blood samples were stored at -80 °C and used for DNA isolation via the “Plus XL manual kit” (LGC). DNA methylation data in samples collected at T0 (*n* = 1,011, subgroup selected for participation at T1) and T1 (*n* = 1,052) were obtained using the “Infinium MethylationEPIC” array, version 1 (Illumina, Inc., USA). Raw data were processed using the “Bigmelon” package in R [19]. Data processing and quality control procedures are described in detail elsewhere. In our main analyses, we investigated “DunedinPACE” calculated as set out in the original publication by Belsky and colleagues [20]. As additional analyses, all main models were also calculated using Horvath’s clock [21], Hannum’s clock [22], PhenoAge [23], GrimAge [24], as well as the principal component (PC) version of these clocks [25]; and finally the 7-CpG clock [26]. The DNA methylation age acceleration (DNAmAA) of these clocks was calculated as residuals of a linear regression of DNA methylation age (DNAmA) on chronological age adjusted for leukocyte cell counts (neutrophils, monocytes, lymphocytes, and eosinophils) and used in the analyses. Further details on how these clocks were calculated in BASE-II can be found elsewhere [18, 27, 28].

#### DNAmGDF15

As directly measured GDF15 is not available in BASE-II, the DNA methylation-derived GDF15 (DNAmGDF15) was estimated from Illumina MethylationEPIC array data using the algorithm published by Lu and colleagues [24].

### Mortality

Information about the BASE-II participants’ mortality status (alive/deceased) is regularly obtained from the Berlin city registry and death certificates of deceased participants are requested from the relevant authorities. Since it is documented that the information in death certificates in Germany can be inconsistent (e.g. ref [29]) they were evaluated by a group of four BASE-II scientists, all of whom are physicians or biologists. Consensus agreements were reached to clarify the causality of direct, indirect and accompanying causes of death as recorded on the mortality records according to the International Statistical Classification of Diseases and Related Health Problems-10 (ICD-10) and the underlying cause of death. For the current analyses, death certificates with information about the cause of death were available for *n* = 180 participants.

### Confounders

Chronological age was calculated as time in years between the participants´ date of birth and date of examination at T0 and T1. Alcohol intake in g/d was assessed via a validated food frequency questionnaire [30]. Smoking behavior was assessed in one-on-one interviews as packyears. To adjust for physical activity, the first question of the Rapid Assessment of Physical Activity (RAPA [31]) questionnaire was used, which showed strong association with accelerometric activity measures available in a subgroup of BASE-II participants at T1 [32]. Genetic ancestry was quantified by the first four principal components from a principal component analysis on genome-wide SNP genotyping data.

### Imputation

All biomarkers, mortality (status, time-to-event, and cause of death) as well as confounders were included in the imputation procedure. To reduce skewness, CRP concentrations were log-transformed using the natural logarithm. IL-6 concentrations were log-transformed after adding a constant of one (log(IL-6 + 1)) to accommodate zero values. All laboratory values underwent outlier exclusion to check for possible measurement errors. Specifically, outliers were defined as values that differed more than 4*SD from the mean. The number of excluded observations was < 0.6% for all biomarkers. Multiple imputations were performed using R’s mice package (10 datasets, 20 iterations, method = “pmm”). A prediction matrix was produced using the *quickpred*-function with a forced inclusion of sex and chronological age at T0. Mortality variables (status, time-to-event, and cause of death) were used as donors but were not imputed. Imputation results were evaluated by comparing the distribution of observed and imputed values in histograms and density plots. Algorithm convergence was checked visually. Dichotomization and definition of groups based on the application of cut-offs applied to continuously-measured variables was done for each imputed dataset individually to guarantee internal consistency within all datasets.

### Statistical analysis

To allow easy comparison of effect sizes between biomarkers on different scales, all continuous variables were z-transformed prior to statistical analysis. This was done for each variable in each imputed dataset individually. All statistical analyses were carried out, and all figures were drawn using the statistical computation software R, version 4.4.1. Descriptive statistics were calculated by the *CreateTableOne*-function (tableone package) using the first imputed dataset as well as the original, not-imputed dataset (Table 1, Supplementary Tables 4–7). Cox proportional hazard regression models were calculated using the *coxph*-function with time from biomarker measurement as underlying time scale (survival package). In line with recommendations on the evaluation of biomarkers of aging [2], unadjusted (model 0) as well as adjusted regression models were calculated. Model 1 was adjusted for age and sex, and model 2 was adjusted for age, sex, alcohol consumption (g/d), smoking (packyears), physical activity (RAPA question 1), and genetic ancestry. As sensitivity analysis, a third adjustment model including BMI, socioeconomic status, morbidity, and polypharmacy in addition to the variables of model 2 was calculated (model 3), and results are shown in the Supplementary Material. Hazard ratios (HRs) and 95% confidence intervals (CIs) were estimated. Cause-specific Cox proportional hazards regression models were calculated to examine associations between biomarkers and cause-specific mortality outcomes in the presence of competing risks. Deaths from known causes other than the cause of interest in the respective cause-specific analysis were treated as competing events and were censored at the time of occurrence. Participants who were alive at the time of the last mortality update were censored at that time. Mortality models investigating all available biomarkers at T0 in a joint model were restricted to the set of participants that were also part of T1 due to the limited availability of DNAm data at T0. To account for delayed entry and avoid immortal time bias, the respective Cox proportional hazard regression models incorporated left truncation, with each participant entering the risk set at their individual follow-up time.

**Table 1:** Descriptive statistics of participants from BASE-II from the first imputed dataset. Due to limited availability at T0, values measured at T1 are shown for methylation derived variables (epigenetic clock, DNAmGDF15) and IGF-1. Original observations are shown in Supplementary Tables 5 and 7

Variables	Level	Alive			Dead		
		*n*	Mean/*n* (%)	SD	*n*	Mean/*n* (%)	SD
Chron. Age (years, T0)		1355	68.3	3.4	316	70.7	4.2
Chron. Age (years, T1)		993	75.5	3.7	90	77.3	4.4
Sex	men		621 (45.8)			188 (59.5)	
	women		734 (54.2)			128 (40.5)	
CRP (mg/L, T0)		1355	2.0	3.0	316	2.2	3.0
IL-6 (pg/mL, T0)		1355	2.6	2.9	316	3.3	3.8
Muscle mass (BMI-standardized ALM, T0)		1355	0.8	0.2	316	0.8	0.2
Hand grip strength (HGS, kg, T0)		1355	34.0	9.8	316	34.3	9.4
Gait speed (s, T0)		1355	3.6	0.7	316	3.8	0.9
Cognitive Health (DSST, T0)		1355	44.8	8.2	316	42.5	9.5
Blood pressure (systolic, mmHg, T0)		1355	143.1	18.8	316	145.9	18.8
Insulin-like growth factor (IGF-1, ng/mL, T1)		993	110.6	35.0	90	107.5	41.1
Epigenetic clock (DunedinPACE, T1)		993	1.1	0.1	90	1.1	0.1
GDF15 (DNAmGDF15, T1)		993	1.157.0	122.1	90	1.173.1	138.0
Muscle strength (T0)	Not impaired		1294 (95.5)			284 (89.9)	
	Impaired		61 (4.5)			32 (10.1)	
Timed-Up-and-Go (TUG, T0)	Not impaired		1217 (89.8)			262 (82.9)	
	impaired		138 (10.2)			54 (17.1)	
Standing balance test (Tinetti Test part1, T0)	No impaired balance		1208 (89.2)			254 (80.4)	
	Possibly impaired balance		147 (10.8)			62 (19.6)	
Frailty (Fried frailty phenotype), T0)	Not frail		963 (71.1)			178 (56.3)	
	Frail		392 (28.9)			138 (43.7)	
Alcohol (g/d, T0)		1355	14.8	17.8	316	15.4	21.4
Smoking (packyears, T0)		1355	9.7	17.1	316	13.2	19.4
Physical Activity (inactive, T0)	no		1239 (91.4)			279 (88.3)	
	yes		116 (8.6)			37 (11.7)	

The *with*-and *pool*-function (mice package) were used to calculate the models individually in each of the imputed datasets and subsequently pool the results employing Rubin’s rules [33]. The proportional hazard assumption was tested in the first imputed dataset using the *cox.zph*-function and by visually inspecting the Schoenefeld residuals plotted over time using survminer’s *ggcoxzph*-function. The pooled C-index across all imputed datasets was calculated using the *cindex*-function (miceafter package). The minimal biomarker model with the best performance in predicting mortality in this dataset was selected using the *stepwise*-function from the StepReg package (strategy = “subset”, metric = “AICc”, sle = 0.15). Because methylation data at baseline were only available for the participants who were also part of the follow-up assessment, the regression models including all available markers at baseline were restricted to this subgroup (n = 1,083). To compare the value added by the biomarkers with respect to discriminate mortality to a basic clinical model, Cox regression models, including only the confounders of model 1 and 2 (no biomarker of aging), were calculated as well. To illustrate differences in the survival probability between groups stratified by the investigated biomarkers, Kaplan-Meier curves were calculated from the first imputed dataset using the *ggsurvplot*-function (survminer package). If no validated cut-off values were available, continuously scaled variables were dichotomized by median split to be investigated in Kaplan-Meier curves with values ≤ 50th quantile grouped as “lower 50%” and values > 50th percentile grouped as “upper 50%”. Due to established sex differences, this procedure was conducted separately for women and men for HGS. Statistical inference was based on two-sided 95% confidence intervals, corresponding to a nominal α-level of 0.05. Associations were considered statistically significant if the confidence interval did not include the null value. Given the a priori selection of biomarkers and the comparative nature of the analyses, no formal correction for multiple testing was applied to avoid an increase in type II error.

## Results

### Study population

**Fig. 1:**
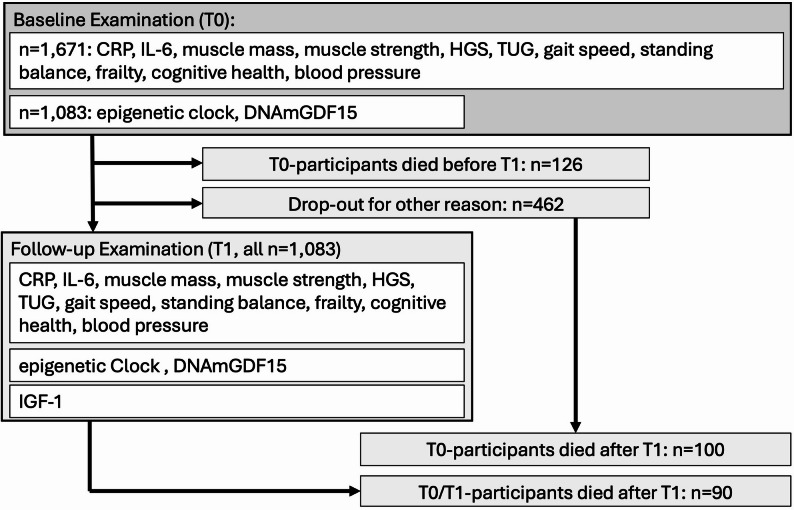
Flow chart of the availability and sample size for biomarkers at baseline (T0) and follow-up (T1) examination investigated. DNA methylation data at T0 was only available for participants who were also part of T1. IGF-1 was only available at T1

A total of 1,671 participants aged 60 years and older were examined during the baseline medical assessment (T0, between 2009 and 2014, mean age = 68.8 years, SD = 3.7 years, 51.6% women). On average 7.4 years later, 1,083 participants were followed up as part of the GendAge study (T1, mean age = 75.6 years, SD = 3.8 years). As of October 2025, a total of 316 deaths had been recorded. Of these, 126 occurred between the baseline and T1 examinations, and 190 occurred after T1. Among those who died after T1, 90 had participated in both the baseline and T1 examinations (Fig. 1). Descriptive statistics of the investigated biomarkers are displayed in Table 1 and Supplementary Tables 4–7. Correlation was generally low between biomarkers (r<|0.4|, Supplementary Fig. 1). As of October 2025, the cause of death is known from death certificates in a subset of 180 participants. The most frequent causes of death were neoplasms (ICD-10: C00-D48, *n* = 78, 43.3%) followed by diseases of the circulatory system (ICD-10: I00-I99, *n* = 46, 25.6%) and diseases of the nervous system (ICD-10: G00-H95, *n* = 18, 10%). All other individual causes of death occurred in 5% of all cases or less and were not investigated separately in this study (Supplementary Table 3).

### Association between mortality and individual biomarkers of aging

**Fig. 2:**
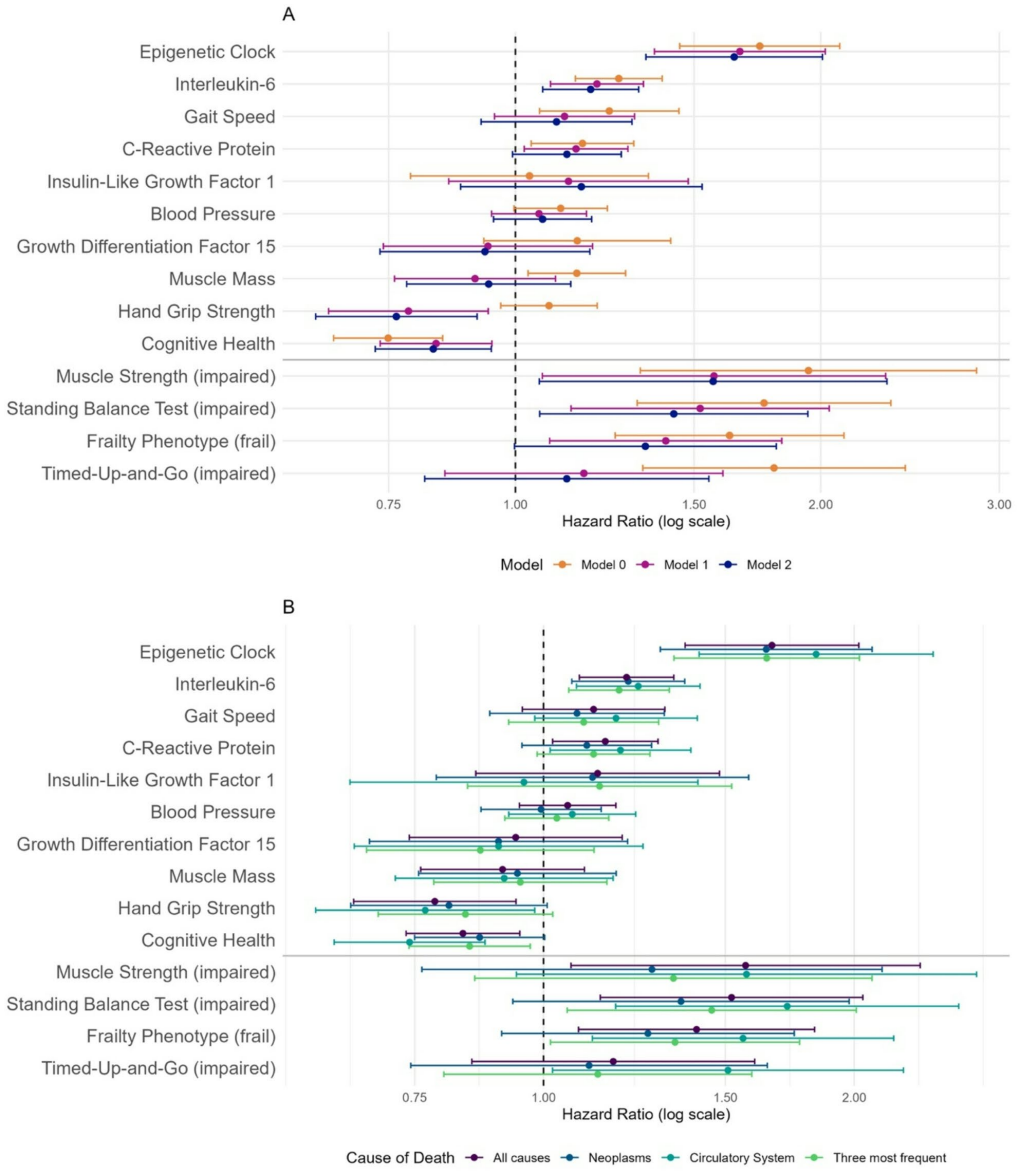
**A**: Forest plot of results from Cox proportional hazard regression analysis of mortality according to biomarkers of aging. All biomarkers were measured at T0 (*n* = 1,671 participants, *n* = 316 deaths) except for DNA methylation-derived variables (epigenetic clock (DunedinPACE) and DNAmGDF15) and IGF-1, which were measured at T1 (*n* = 1,083 participants, *n* = 90 deaths) on average 7.4-years later. All continuously scaled variables were normalized to allow direct comparison of effect sizes across different scales. Model 0: unadjusted; Model 1: age, sex; Model 2: Model 1 + alcohol, smoking, physical activity, genetic heredity (PC1-PC4). **B**: Forest plot of results from an age- and sex-adjusted Cox proportional regression analysis (model 1) of mortality according to biomarkers of aging in subgroups defined by cause of death as obtained from the death certificates in a subgroup of BASE-II participants (*n* = 180). All biomarkers were measured at T0 except for DNA methylation-derived variables (epigenetic clock (DunedinPACE), DNAmGDF15) and IGF-1 which were measured at T1. The values of the all-cause group are shown for easy comparison with subgroup results and are identical to values shown in A (model 1)

**Fig. 3:**
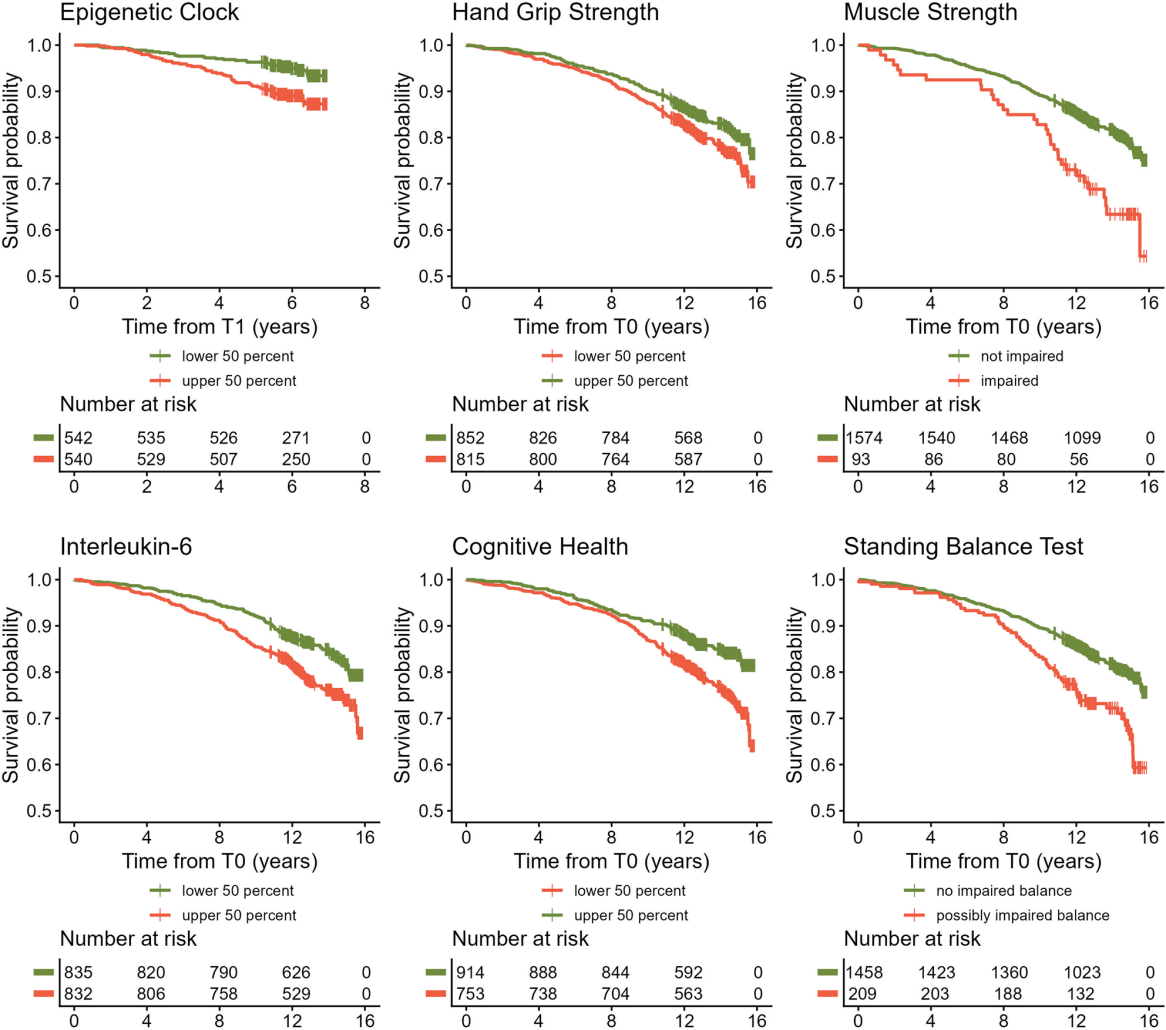
Kaplan-Meier analysis of biomarkers with the strongest association with mortality. Due to an overall low mortality rate in the sample, the y-axis limits were adjusted to improve readability of the figure. All biomarkers were assessed at T0 (*n* = 1,671 participants, *n* = 316 recorded deaths) except for the epigenetic clock which was derived from sample collected as part of T1 (*n* = 1,083 participants, *n* = 90 recorded deaths)

To investigate associations between the 14 biomarkers identified in the consensus procedure published by Perri and colleagues [5] and mortality, Cox proportional hazard regression models were calculated. DNAm derived variables at T0 were only available in the subgroup of participants who also were part of T1. Thus, all investigated biomarkers were assessed at T0, except for DNAm derived variables (to avoid survivorship bias in analyses not adjusted for left truncation) and IGF-1 (not available at T0) for which the data assessed at T1 was examined. A statistically significant association with mortality was found in the age- and sex-adjusted regression models (model 1) for all biomarkers except IGF-1, gait speed, blood pressure, DNAmGDF15, muscle mass, and TUG. After additionally adjusting for alcohol consumption, smoking (packyears), physical activity and genetic ancestry (model 2) the epigenetic clock (DunedinPACE), IL-6, cognitive health, HGS, muscle strength and standing balance remained statistically significantly associated with mortality (Fig. 2A, Supplementary Table 8). Kaplan-Meier curves of the biomarkers with the strongest association with mortality are displayed in Fig. 3. In sex-stratified subgroup analyses, generally similar effect sizes compared to the whole group were observed with stronger effects in the subgroup of men (Supplementary Table 8). For example, whereas no association was found between HGS and mortality in women, we observed a fairly strong association with mortality in men (HR: 0.68, 95%CI: 0.54–0.84). This sex difference was statistically significant (p-value for biomarker-sex-Interaction: *p* ≤ 0.049, Supplementary Table 8). We repeated all regression models of biomarkers at T0 with available values measured at T1 as sensitivity analyses to enable a direct comparison for variables which were only assessed at T1 (Supplementary Table 9). In addition, a Cox regression model further adjusted for body mass index, socioeconomic status, morbidity index, and polypharmacy (model 3) was calculated as a sensitivity analysis (Supplementary Tables 8–11). Compared with model 2, only small numerical differences in the estimated hazard ratios were observed and the inclusion of additional confounders did not change the interpretation of the results except for the association between mortality and muscle strength at T0 (Supplementary Table 8), and the association with cognitive health at T1 in the male subgroup (Supplementary Table 9), for which the association was not statistically significant after additional adjustment anymore.

### Analyses stratified by cause of death

To investigate cause of death as a possible effect measure modifier, subgroup analyses were conducted on participants who died from diseases of the circulatory system, neoplasms, and the three most frequent causes of death (diseases of the circulatory system, neoplasms, and diseases of the nervous system; Supplementary Table 3). The epigenetic clock (DunedinPACE) and IL-6 remained statistically significantly associated with mortality among all subgroups (Fig. 2B, Supplementary Tables 10, 11). Across all biomarkers, the strongest associations were found in the subgroup of participants who died from diseases of the circulatory system except for DNAmGDF15 and IGF-1 which was most strongly associated with death due to the three most frequent causes of death. All investigated biomarkers were assessed at T0 except for the DNAm derived markers (epigenetic clock, DNAmGDF15) and IGF-1 for which the data collected at T1 were examined. Whenever available, we repeated regression models of biomarkers at T0 with values measured at T1 as sensitivity analyses (Supplementary Table 11).

### All biomarkers of aging and the best minimal set of biomarkers to predict mortality

Next, a Cox regression model including all available biomarkers of aging at T0 (Table 2, “Full Model”) was calculated to evaluate the joint ability of all biomarkers (except IGF-1, which was not measured at T0, and muscle strength to avoid redundancy with HGS) to predict mortality. Analyses were limited by the availability of baseline DNAm data, which was present for the subgroup of 1,083 participants who were also part of the follow-up examination (*n* = 90 recorded deaths). The full Cox regression model had a C-index of 0.65. Including the confounder variables of model 1 (C-index: 0.70) and model 2 (C-index: 0.72) increased the discrimination of the Cox regression model further by up to 7.2 percentage points. Adding all available biomarkers of aging to a Cox regression model including only the confounding variables of model 1 and 2 increased the C-index by 3.9 and 4.1 percentage points (Supplementary Table 12). As a sensitivity analysis, the full model was also calculated using muscle strength instead of HGS (Supplementary Table 12, Supplementary Table 13), which resulted in small numerical changes in the effect estimates but did not alter the overall interpretation of results.

**Table 2:** Cox proportional hazard regression with all (“Full Model”) or a minimal subset (“Minimal Model”) of markers. All markers in this analysis were assessed at baseline. Only participants who were also part of T1 are included in this analysis (*n* = 1,083, *n* = 90 deaths). Left truncation of the data was included in the regression analysis. As no IGF-1 values are available at T0, this biomarker was not part of this model. To avoid redundancy, muscle strength, which was derived from HGS, was not included in the full model. As a sensitivity analysis, a full model considering muscle strength instead of HGS is shown in Supplementary Table 13

Biomarker	HR	lower 95% CI	higher 95% CI
**Full Model**			
Epigenetic clock	1.40	1.11	1.75
DNAmGDF15	1.01	0.81	1.25
CRP	0.96	0.75	1.23
IL-6	1.11	0.89	1.39
Muscle mass	1.28	0.95	1.73
Hand grip strength	0.91	0.67	1.22
Gait speed	1.04	0.77	1.42
Cognitive Health	0.92	0.73	1.16
Blood pressure	1.03	0.83	1.26
Timed-Up-and-Go (impaired)	1.38	0.65	2.93
Standing balance test (impaired)	1.57	0.85	2.90
Frailty phenotype (frail)	1.00	0.53	1.86
**Minimal Model**			
Epigenetic clock	1.45	1.19	1.77
Muscle mass	1.21	0.98	1.49
Standing balance (impaired)	1.72	0.98	3.01

To determine the best subset of biomarkers for predicting mortality in BASE-II, a feature-selection approach was applied, considering all possible combinations of variables in the Cox regression models. The best-fitting model included the epigenetic clock (DunedinPACE), muscle mass, and performance in the standing balance test. A Cox regression model with these biomarkers provided only slightly decreased discriminatory power (C-index: 0.63) compared to the full model (C-index: 0.65) with only a fraction of the biomarkers. Adding this minimal set of variables to the Cox regression models including variables in model 1 and 2, increased their discrimination both by 2.5 percentage points (Supplementary Table 12).

## Discussion

In this study, we used data obtained from 1,671 participants of the BASE-II to compare 14 biomarkers of aging which were selected through a comprehensive consensus procedure and were previously published by Perri and colleagues [5] as outcome variables for anti-aging intervention studies. This is the first study that investigates and compares all 14 biomarkers in the same, well-described sample of study participants. The analyses revealed individual strengths and weaknesses of these biomarkers concerning mortality. Epigenetic clock (DunedinPACE), IL-6, HGS, cognitive health, muscle strength, and standing balance test were statistically significantly associated with mortality in confounder adjusted models and had effect sizes generally in the same range as reported in the literature (Supplementary Table 1). We note that the high predictive value of the standing balance test corroborates earlier analyses of the Berlin Aging Study (the predecessor study of BASE-II) [34]. The epigenetic clock (DunedinPACE) showed the strongest association, and this was also observed in subgroup analyses stratified by cause of death. These results are in line with the results reported in the original publication of DunedinPACE investigating this biomarker’s association in the Normative Aging Study (*n* = 771 men, mean age: 77 years, HR: 1.26, 95%CI 1.14–1.40) and Framing Heart Study (FHS, *n* = 2471, 54% women, mean age 66 years, HR 1.65, 95%CI 1.51–1.79). Subsequent studies confirmed these initial results and reported 1.23-fold increase in HR (95% CI 1.05–1.44) per 1-SD increase in DunedinPACE in participants of the Finnish Twin Study on Aging (FITSA) [35]. Even in a younger cohort of 2,216 veterans (21% women, mean age 37.4 years), increased DunedinPACE was associated with a higher mortality hazard (HR 1.38; 95% CI 1.12–1.72) [36]. While the comparability of results might be limited due to differences in the underlying source population or sex distribution, they all suggest DunedinPACE as a valuable biomarker with consistent mortality association. In additional analyses, we compared DunedinPACE with other established epigenetic clock algorithms. After confounder adjustment, the one other epigenetic clock showing a statistically significant association with mortality was GrimAge (Supplementary Fig. 2). This association is not surprising as GrimAge was trained to predict mortality [24].

Intriguingly, in cause-specific subgroup analyses, all biomarkers (except DNAmGDF15 and IGF-1) were most strongly associated with cardiovascular deaths (Fig. 2B). These results might partly be explained by associations with cardiovascular disease which are well-established, for example, for frailty [37]. This known relationship might also be the reason for the observed association of individual frailty components, such as HGS and the TUG, with cardiovascular death. These results confirm previous findings of a strong association between TUG and cardiovascular mortality [38, 39] as well as of an unadjusted association of all-cause mortality and frailty based on data from an earlier mortality update in BASE-II [40]. On the other hand, after adjusting for confounding variables, CRP, gait speed, IGF-1, blood pressure, muscle mass, DNAmGDF1, frailty and TUG were not associated with all-cause mortality. However, the lack of a (strong) association with mortality should not necessarily be interpreted as evidence against the validity of these biomarkers, as limited statistical power, sample size, or age-range constraints may obscure true underlying effects. Moreover, as people spend more time living with disease in later life, markers that distinguish healthy from unhealthy aging remain valuable, even if they are not associated with mortality. Also, the instruments/methods used in this study to assess the biomarkers suggested by Perri and colleagues are often only one of numerous possible options to derive the respective variables. For example, although we used the DSST to assess *cognitive health*, this construct can be operationalized using numerous other cognitive measures and tests. Further studies evaluating these 14 biomarkers of aging in the same cohort, not only in the context of mortality but also other age-associated outcomes, such as healthspan, chronic diseases and quality of life are needed. Such analyses would help to clarify the biomarkers’ individual strengths beyond mortality prediction.

In a final step, we investigated the joint ability of constellations of the pre-selected biomarkers of aging to predict mortality. Overall, the correlation among biomarkers was weak (*r* ≤ ∣0.4∣, Supplementary Fig. 1). Comparing a model containing all biomarkers of aging available at T0 (except muscle strength to avoid redundancy with HGS) to a model containing a minimal subset (epigenetic clock (DunedinPACE), muscle mass, and standing balance test) the full cluster of markers seemed to provide only limited additional value of 3.9 and 2.5% points, respectively, as compared to a basic model including age and sex. This implies that despite only weak inter-correlations, the markers seem to convey largely overlapping prognostic information. Thus, although these markers are useful for conceptualizing biological aging, they may offer limited incremental value for individual-level mortality prediction. Moreover, the selection of variables in the minimal model is by no means final. Using a different selection procedure or a different study sample, which stems from a different source population and/or differs in other aspects (such as age, morbidity, demographic variables) might identify different sets of optimal variables. Also, in this study, the biomarker–mortality relationship was assessed using Cox regression models, a well-established approach that facilitates comparison with prior studies which most often use the same methodology. Alternatives such as machine learning models could uncover additional associations or different minimal biomarker sets. However, previous research was not able to show a consistent advantage of such methods over Cox regression analyses [41]. This seems to be especially true for instances where the number of events is rather small (as is the case here) since machine learning methods often require large datasets to produce stable estimates [41]. The investigation of mortality prediction using aging markers therefore remains an intriguing topic for future research.

Whereas the consensus on the 14 markers of aging was an important and highly valuable contribution to the field, the identified markers are only qualitatively described in the publication by Perri and colleagues. Thus, for most variables, no specific standardized instruments or cut-off values (if applicable) are defined. Additional studies using different methods to assess the proposed biomarkers of aging are needed, thereby providing information on which instruments are best suited as outcome measures in anti-aging intervention studies. Furthermore, it is likely that promising biomarkers for mortality prediction were not included in the final set of consensus-based biomarkers of biological aging, whether because they are less well known, costly or difficult to measure, or were too recently developed to have been thoroughly investigated. As the primary aim of this study was to compare the 14 suggested biomarkers, other potentially informative predictive markers were not examined here. Accordingly, whereas the work by Perri and colleagues [5] represents an important and highly valuable contribution to the effort to harmonize the evaluation of biomarkers of aging, it does not rule out the possibility that the list may be amended if new promising biomarkers emerge or if existing biomarkers are shown to perform better.

Strengths of this study include the availability of all 14 biomarkers of aging in the same large sample of older participants. Follow-up of up to 16 years provides a sufficient period to assess mortality for variables assessed during the BASE-II baseline assessment (T0). High quality of the cause of mortality data was ensured by an internal review procedure including an interdisciplinary team of scientists. By using the multiple imputation approach, all variables are compared within the same group of participants, which increases the comparability of effect sizes between variables. Nonetheless, this study has several limitations. First, data availability with respect to some biomarkers (DNAm derived variables, IGF-1) at baseline was limited. Thus, the follow-up time as well as the mean age differs between regression models investigating these biomarkers and all others. Second, for technical reasons, the cause of death was only available for a subgroup of participants and future studies with fully available information on cause of death are needed. Fourth, no adjustment for multiple testing was made. Thus, the findings should be interpreted cautiously, and external replication of the comparative evaluation of biomarkers is needed to confirm the reported results. Fifth, because BASE-II participants have above-average health compared to general population [11], the generalizability of our findings to the underlying source population may be limited and it is possible that true effect sizes are underestimated. For example, as a sensitivity analysis, we assessed the frailty phenotype at follow-up using a stricter definition of frailty (requiring three or more components fulfilled instead of a minimum of one to be classified as frail). Substantially stronger effect sizes were observed (Supplementary Table 14), suggesting that in a cohort with overall poorer health, stronger associations might be expected. Finally, as the analyzed data are observational in nature, it was only possible to investigate the predictive ability of the examined biomarkers and we note that our data do not allow to infer on any causal relationship with mortality. This question can only be addressed in studies that specifically investigate interventions changing individual biomarkers.

## Conclusions

Among the 14 consensus biomarkers suggested by Perri and colleagues, hand grip strength, IL-6, standing balance, cognitive health, and epigenetic clock (DunedinPACE), were associated with mortality. Epigenetic clock (DunedinPACE) showed the strongest and most consistent association with mortality. A minimal joint biomarker model including muscle mass, standing balance and epigenetic clock (DunedinPACE) showed similar accuracy (C-index = 0.63) as the full model including all biomarkers (C-index = 0.65) suggesting the presence of a substantial degree of overlap between the investigated biomarkers with respect to mortality prediction. Further studies validating the suggested markers within additional single cohorts are needed to explore further and confirm biomarkers of human aging, particularly in diverse populations [42].

## Supplementary Information

Below is the link to the electronic supplementary material.

Supplementary Material 1

Supplementary Material 2

## Data Availability

Due to concerns for participants privacy as well as data protection regulations, BASE-II raw data cannot be made publicly available. Interested investigators are invited to contact the scientific coordinator of BASE-II, Ludmila Müller (lmueller@mpib-berlin.mpg.de), to obtain raw data access for project-specific analyses under an individually negotiated data use agreement. Additional information can be found on the BASE-II website: https://www.base2.mpg.de/7549/data-documentation.

## Abbreviations

ALM: Appendicular lean mass
BASE-II: Berlin Aging Study II
BMI: Body Mass Index
CRP: C-reactive protein
DBS: Dried blood spots
DNAm: DNA methylation
DNAmAA: DNA methylation age acceleration
DNAmGDF15: Growth-differentiating factor-15, DNA methylation derived
DSST: Digit Symbol Substitution Test
ELISA: Enzyme-linked Immunosorbent Assay
FP: Frailty phenotype
HGS: Hand grip strength
hsCRP: High-sensitivity C-reactive protein
IGF-1: Insulin-like growth factor 1
IL-6: Interleukin-6
PBS: Phosphate-buffered saline
PC: Principal component
RAPA: Rapid Assessment of Physical Activity
TUG: Timed-Up-and-Go
