# Supplementary material for: Comparing fourteen consensus biomarkers of aging: epigenetic pace of aging as the strongest predictor of mortality in BASE-II
Source: Biomark Res. 2026 Mar 6;14:37. doi: 10.1186/s40364-026-00909-z (PMC13063672; doi:10.1186/s40364-026-00909-z)
Supplement: Supplementary file 1 — Supplementary Material 1 [file 40364_2026_909_MOESM1_ESM.docx]

**Comparing Fourteen Consensus Biomarkers of Aging: Epigenetic Pace of Aging as the Strongest Predictor of Mortality in BASE-II**

Valentin Max Vetter, MD, MSc^1^, Marit Philine Junge, MSc^2^, Christian A. Drevon, MD, PhD^3,4^, Thomas E. Gundersen, PhD^3^, Jan Homann, MSc^5^, Christina M. Lill, MD, MSc^5,6^, Ulman Lindenberger, PhD^7,8^, Graham Pawelec, PhD^9,10^, Lars Bertram, MD^2^, Denis Gerstorf, PhD^11^, Ilja Demuth, PhD^1,12^

1 Charité – Universitätsmedizin Berlin, corporate member of Freie Universität Berlin and Humboldt-Universität zu Berlin, Department of Endocrinology and Metabolic Diseases (including Division of Lipid Metabolism), Biology of Aging working group, Augustenburger Platz 1, 13353 Berlin, Germany

2 Lübeck Interdisciplinary Platform for Genome Analytics (LIGA), University of Lübeck, Lübeck, Germany.

3 Vitas Ltd. Oslo Science Park, Oslo, Norway

4 Department of Nutrition, Institute of Basic Medical Sciences, Faculty of Medicine, University of Oslo, Oslo, Norway.

5 Institute of Epidemiology and Social Medicine, University of Münster, Münster, Germany

6 Ageing Epidemiology Research Unit, School of Public Health, Imperial College, London, UK

7 Center for Lifespan Psychology, Max Planck Institute for Human Development, Berlin, Germany

8 Max Planck UCL Centre for Computational Psychiatry and Ageing Research, Berlin, Germany, and London, UK

9 Institute of Immunology, University of Tübingen, Tübingen, Germany

10 Health Sciences North Research Institute, Sudbury, ON, Canada

11 Department of Psychology, Humboldt University Berlin, Berlin, Germany.

12 Charité - Universitätsmedizin Berlin, BCRT - Berlin Institute of Health Center for Regenerative Therapies, Berlin, Germany

Supplementary Methods

*Confounders*

Morbidity was assessed using a modified version (Meyer et al. 2016) of Charlson’s Comorbidity Index (Charlson et al. 1987). The following diseases were included in the calculation of the index: myocardial infarct, congestive heart failure, peripheral vascular disease, cerebrovascular disease, ulcer disease, dementia, chronic pulmonary disease, connective tissue disease, mild liver disease, diabetes, diabetes with end-organ damage, renal disease, hemiplegia, lymphoma, leukemia, any tumor, and moderate to severe liver disease. Depending on the disease, weights between 1 and 3 were assigned in accordance with the original publication.

Participants’ medications were assessed during the one-on-one interview with study personnel. Participants were asked to bring all regularly used medicine packages as well as their medication plan. Study personnel reviewed these materials and recorded a comprehensive medication history, including indications, dosages, start dates, and side effects. The number of regularly used medications was calculated by summing all prescribed, over-the-counter, and traditional drugs reported by participants (excluding on-demand medications). Polypharmacy defined as the regular use of five or more drugs (Toepfer et al. 2019) which is line with previous BASE-II publications (König et al. 2017, König et al. 2018).

The variable years of education was used as a proxy for the socioeconomic status.Body Mass Index (BMI) was calculated as kg/m^2^ from height and body weight recorded with an electronic SECA measuring station (model 763, Germany).

Supplementary Table 1: Non-exhaustive overview of selected publications reporting on the association between the investigated biomarkers of aging and mortality.

| Marker | Publication | Sample Size  Number of Deaths | Instrument | Results |
| --- | --- | --- | --- | --- |
| insulin-like growth factor 1 | (De Giorgi et al. 2022) | N=337 (19.3% women)  N=41  patients with moderately or severely HFrEF, enrolled in the T.O.S.CA. registry | IGF-1 | Cox Regression: HR: 0.42, 95%CI:0.23-0.77 |
|  | (Chen et al. 2023) | N=685 (28% women)  N=208  Patients with Chronic Kidney Disease | IGF-1 | Competing risk regression:  high HGS + Low IGF-1: ref. group (SHR: 1)  high HGS + High IGF-1: SHR: 1.1, 95%CI: 0.66-1.83  low HGS + High IGF-1: SHR: 2.21, 95%CI:1.37-3.55  low HGS + low IGF-1: 2.76, 95%CI: 1.79-4.27 |
|  | (Rahmani et al. 2022) | Meta-Analysis of 19 studies  N=30,876 | IGF-1 | random effects model with a restricted maximum  likelihood heterogeneity variance estimator: high vs. low IGF-1: HR:0.84, 95%CI: 0.68–1.05  U-shaped relationship:  low vs. middle: IGF-1: HR: 1.33, 95%CI: 1.14–1.57)  high vs. middle IGF-1: HR:1.23, 95%CI: 1.06–1.44 |
| growth-differentiating factor-15 | (Daniels et al. 2011) | N=1391 (61% women)  N=436  Rancho Bernardo Study participants | GDF-15 | Cox proportional hazard regression: HR per SD log_10_ units: 1.5, 95%CI:1.3-1.8) |
|  |  | N=14577  N= 1067  Stabilization of Atherosclerotic Plaque by Initiation of Darapladib Therapy Trial (STABILITY) | GDF-15 | Cox proportional hazard regression: Highest vs. lowest quartile of GDF-15:HR 1.85, 95%CI: 1.53-5.41 |
| high sensitivity C-reactive protein | (Li et al. 2017) | Meta-Analysis  N=83,995 | hsCRP | Highest vs. lowest category: RR:1.75, 95%CI: 1.55–1.98 |
|  | (Boekholdt et al. 2006) | N=3272  N=987  The EPIC-Norfolk prospective population study 1993–200 | CRP | Confounder adjusted logistic regression: highest vs. lowest CRP-Quartile OR: 2.92, 95%CI: 1.83–4.67 |
| interleukin-6 | (Lee et al. 2012) | N=1843  N = 978  The Rancho Bernardo Study | IL-6 | Cox proportional hazard regression, confounder adjusted: HR:1.48, 95%CI: 1.33–1.64 |
|  | (Puzianowska-Kuźnicka et al. 2016) | N=3750 (47.8% women)  1-year mortality rate: 6.6%  PolSenior study | IL-6 | Cox proportional hazard regression, unadjusted: HR: 1.077 per each pg/mL, 95%CI:1.068–1.086 |
| muscle mass | (de Santana et al. 2021) | Meta-Analysis  N=10028 | appendicular skeletal muscle mass index (ASMI) | SMD between dead and living participants: ASMI SMD = −0.18, 95%CI: −0.23 to −0.12 |
|  | (Abramowitz et al. 2018) | N=11687  N=1819  National Health and Nutrition Examination Survey 1999–2004 | appendicular skeletal muscle mass index (ASMI) | Cox proportional hazard regression analysis: HR 0.82 per 1 kg/m^2,^ 95%CI:0.73–0.92 |
| muscle strength | (Jochem et al. 2019) | Meta-Analysis  N=39,852 | knee extension strength, knee flexion strength, hand grip strength, quadriceps isometric strength, quadriceps maximal voluntary contraction force, Bench press leg press, Medical Research Council scale for muscle strength | Random-effects meta-regression: Lowest vs. highest category: HR1.80, 95%C: 1.54–2.10 |
| hand grip strength | (García-Hermoso et al. 2018) | Meta-Analysis:  N=1,907,580  N= 63,087 | Hand Grip Strength | Cox proportional hazard regression: HR=0.69; 95% CI, 0.64-0.74 |
|  | (Chua et al. 2020) | N=13,789  N= 533 | Hand Grip Strength | Cox proportional hazard regression: lowest vs. highest quartile: HR: 2.05, 95%CI:1.44–2.90 |
| timed “Up & Go” | (Ascencio et al. 2022) | N=427  N=81 | Timed “Up & Go” | Cox proportional hazard regression: HR = 1.05; 95% CI: 1.02–1.09 |
|  | (Chua et al. 2020) | N=13,789  N= 533 | Timed “Up & Go” | Cox proportional hazard regression: lowest vs. highest quartile: HR:3.08, 95%CI: 2.17–4.38 |
| gait speed | (Studenski et al. 2011) | Meta-Analysis  N=34,485  N=17,528 | Gait Speed (distance between 8 ft and 6 m) | HR per 0.1 m/s: 0.88, 95%CI: 0.87-0.90 |
|  | (Rolland et al. 2006) | N=7,250  N=754  older French women enrolled in Epidémiologie de l’ostéoporose (EPIDOS) | 6-m walking speed | Cox proportional hazard regression: highest vs. lowest category: HR: 6.01, 95%CI: 2.81-12.83 |
|  | (Cooper et al. 2010) | Meta-Analysis  N=14 692 | Walking speed | Confounder adjusted, lowest vs. highest quartile: HR 2.87, 95%CI: 2.22 - 3.72 |
| standing balance test | (Cao et al. 2021) | N=5816  N=1530 | modified Romberg Test of Standing Balance on Firm and Compliant Support Surfaces | Cox proportional hazard regression: balance impairment vs. no balance impairment HR: 1.44, 95%CI: 1.23-1.69 |
|  | (Cesari et al. 2009) | N=3024  N=653  Health ABC Study | balance (semi- and full-tandem, and single leg stands each held for 30 seconds) tests | Cox proportional hazard regression, confounder adjusted for <53 seconds vs. ≥53 seconds: HR: 1.35, 95%CI: 1.12 - 1.62 |
| frailty phenotype | (Vermeiren et al. 2016) | Meta-Analysis,  N=150,763 | 29 different frailty instruments | Meta-Analysis of RR and HR together:  Premature mortality: RR 1.83, 95%CI:1.68–1.98 |
|  | (Chang and Lin 2015) | Meta-Analysis,  N=35,538  N=7,994 | Fried’s Frailty Phenotype | Cox proportional hazard regression:   - robust vs. frail: HR: 2.0; 95% CI: 1.73–2.32 - frail vs. pre-frail: HR: 1.48; 95%CI: 1.34–1.63 |
| cognitive health | (Pavlik et al. 2003) | N= 11,444  N= 482  ARIC Study | DSST | Cox proportional hazard regression: HR per 7-point DSST score increment: 0.86, 95%CI: 0.80-0.93 |
|  | (Rosano et al. 2008) | N=3,156  N=704  Cardiovascular Health Study | DSST | Cox proportional hazard regression: HR: 0.77, 95%CI: 0.68–0.87 |
|  | (Adjoian Mezzaca et al. 2022) | N=5,989  National Health and Nutrition Examination Survey | DSST (inverse coded) | Cox proportional hazard regression: HR per 1-SD change in DSST: 1.36, 95%CI: 1.25-1.48 |
| blood pressure | (Satish et al. 2001) | N=12,802  EPESE | Systolic Blood Pressure | Cox proportional hazard regression, confounder adjusted:   - men 65-84 years old: HR per 10 mmHG increase: 1.04, 95%CI: 1.01-1.07 - men >84 years old: HR per 10 mmHG increase: 0.92, 95%CI: 0.86-0.99 |
|  | (Todd et al. 2019) | Meta-Analysis  N=21,906 | Systolic Blood Pressure | Fixed-effect meta analysis, <140mmHG vs. >140mmHG:   - participants with frailty: HR 1.02, 95% CI 0.90 - 1.16 - participants without frailty: HR 0.86, 95% CI 0.77 - 0.96 |
| DNA methylation/epigenetic clocks | (Marioni et al. 2015) | Meta-Analysis  N=4,658  N=862 | Δ_age_ (Horvath clock and Hannum clock) | Cox proportional hazard regression, confounder adjusted:  5-year higher Δ_age_ (Hannum): HR 1.21, 95%CI: 1.14-1.29  5-year higher Δ_age_ (Horvath): HR 1.09, 95%CI: 1.02-1.15 |
|  | (Föhr et al. 2023) | N=395  N=187  Finnish Twin Study on Aging (FITSA) | GrimAge  DunedinPACE | Cox proportional hazard regression, unadjusted:  1-SD increase in GrimAge HR: 1.36, 95%CI: 1.18-1.57  1-SD increase in DunedinPACE HR: 1.23, 95%CI: 1.05-1.44 |

Supplementary Table 2: Overview of variables available in BASE-II, their respective assessment methods and their inclusion in main and sensitivity analyses.

| **Biomarker** | **BASE-II Variable** | **Method** | **Used in** |
| --- | --- | --- | --- |
| **T0** | | | |
| growth differentiation factor 15 (GDF15) | GDF15 | DNAm-predicted, Illumina MethylationEPIC | Main analysis |
| C-reactive protein (CRP) | CRP | Standard Laboratory, immunoturbidimetry | Main analysis |
| Interleukin-6 (IL-6) | Interleukin-6 (IL-6) | Cytometric Bead Array flex kit | Main analysis |
| Muscle Mass | BMI-standardized appendicular lean mass (ALM) | Hologic^®^ QDR^®^ Discovery™ dual-energy X-ray absorptiometry | Main analysis |
| Muscle Strength | HGS cut-off | Sex- and BMI-specific cut-offs for muscle strength on HGS defined by Fried et al. (Fried et al. 2001) | Main analysis |
| Hand Grip Strength (HGS) | HGS | Smedley Dynamometer | Main analysis |
| Timed-Up-and-Go (TUG) | Timed-Up-and-Go (TUG) | Clinical Assessment | Main analysis |
| gait speed | 4m gait speed | time (in s) needed to walk 4 meters | Main analysis |
| standing balance test | Tinetti Mobility Test, part 1 | Clinical Assessment | Main analysis |
| frailty phenotype | Fried’s Frailty Phenotype | As described in (Fried et al. 2001) and (Spira et al. 2015) | Main analysis |
| Cognitive Health | Digit Symbol Substitution Test (DDST) | Neuropsychological Assessment | Main analysis |
| Blood Pressure (BP) | Systolic Blood Pressure (BP) | boso-medicus memory electronic Sphygmomanometer | Main analysis |
| Epigenetic Clock | DunedinPACE | DNAm-predicted, Illumina MethylationEPIC array | Main analysis |
| Epigenetic Clock | DNAmAA Horvath | DNAm-predicted, Illumina MethylationEPIC array | Additional analysis |
| Epigenetic Clock | DNAmAA Hannum | DNAm-predicted, Illumina MethylationEPIC array | Additional analysis |
| Epigenetic Clock | DNAmAA PhenoAge | DNAm-predicted, Illumina MethylationEPIC array | Additional analysis |
| Epigenetic Clock | DNAmAA GrimAge | DNAm-predicted, Illumina MethylationEPIC array | Additional analysis |
| Epigenetic Clock | DNAmAA GrimAge2 | DNAm-predicted, Illumina MethylationEPIC array | Additional analysis |
| Epigenetic Clock | DNAmAA PCHorvath1 | DNAm-predicted, Illumina MethylationEPIC array | Additional analysis |
| Epigenetic Clock | DNAmAA PCHorvath2 | DNAm-predicted, Illumina MethylationEPIC array | Additional analysis |
| Epigenetic Clock | DNAmAA PCHannum | DNAm-predicted, Illumina MethylationEPIC array | Additional analysis |
| Epigenetic Clock | DNAmAA PCPhenoAge | DNAm-predicted, Illumina MethylationEPIC array | Additional analysis |
| Epigenetic Clock | DNAmAA PCGrimAge | DNAm-predicted, Illumina MethylationEPIC array | Additional analysis |
| Epigenetic Clock | DNAmAA 7-CpG | DNAm-predicted, MS-SNuPE | Additional analysis |
| **T1** | | | |
| Insulin-Like Growth Factor 1 (IGF-1) | Insulin-Like Growth Factor 1 (IGF-1) | Dried Blood Spots (VITAS Analytical Services) | Main analysis |
| Growth Differentiation Factor 15 (GDF 15) | Growth Differentiation Factor 15 (GDF 15) | DNAm-predicted, Illumina MethylationEPIC array | Main analysis |
| C-reactive protein (CRP) | CRP | Standard Laboratory, immunoturbidimetry | Main analysis |
|  | hsCRP | Dried Blood Spots (VITAS Analytical Services) | Sensitivity analysis |
| Muscle Mass | BMI-standardized appendicular lean mass (ALM) | Hologic^®^ QDR^®^ Discovery™ dual-energy X-ray absorptiometry | Main analysis |
| Muscle Strength | HGS cut-off | Sex- and BMI-specific cut-offs for muscle strength on HGS defined by Fried et al. (Fried et al. 2001) | Main analysis |
| Hand Grip Strength (HGS) | HGS | Smedley Dynamometer | Main analysis |
| Timed-Up-and-Go (TUG) | Timed-Up-and-Go (TUG) | Clinical Assessment | Main analysis |
| standing balance test | Tinetti Mobility Test, part 1 | Clinical Assessment | Main analysis |
| frailty phenotype | Fried’s Frailty Phenotype | As described in (Fried et al. 2001) and (Spira et al. 2015) | Main analysis |
| Cognitive Health | Digit Symbol Substitution Test (DDST) | Neuropsychological Assessment | Main analysis |
| Blood Pressure (BP) | Systolic Blood Pressure (BP) | boso-medicus memory electronic Sphygmomanometer | Main analysis |
| Epigenetic Clock | DunedinPACE | DNAm-predicted, Illumina MethylationEPIC array | Main analysis |
| Epigenetic Clock | DNAmAA Horvath | DNAm-predicted, Illumina MethylationEPIC array | Additional analysis |
| Epigenetic Clock | DNAmAA Hannum | DNAm-predicted, Illumina MethylationEPIC array | Additional analysis |
| Epigenetic Clock | DNAmAA PhenoAge | DNAm-predicted, Illumina MethylationEPIC array | Additional analysis |
| Epigenetic Clock | DNAmAA GrimAge | DNAm-predicted, Illumina MethylationEPIC array | Additional analysis |
| Epigenetic Clock | DNAmAA GrimAge2 | DNAm-predicted, Illumina MethylationEPIC array | Additional analysis |
| Epigenetic Clock | DNAmAA PCHorvath1 | DNAm-predicted, Illumina MethylationEPIC array | Additional analysis |
| Epigenetic Clock | DNAmAA PCHorvath2 | DNAm-predicted, Illumina MethylationEPIC array | Additional analysis |
| Epigenetic Clock | DNAmAA PCHannum | DNAm-predicted, Illumina MethylationEPIC array | Additional analysis |
| Epigenetic Clock | DNAmAA PCPhenoAge | DNAm-predicted, Illumina MethylationEPIC array | Additional analysis |
| Epigenetic Clock | DNAmAA PCGrimAge | DNAm-predicted, Illumina MethylationEPIC array | Additional analysis |
| Epigenetic Clock | DNAmAA 7-CpG | DNAm-predicted, MS-SNuPE | Additional analysis |


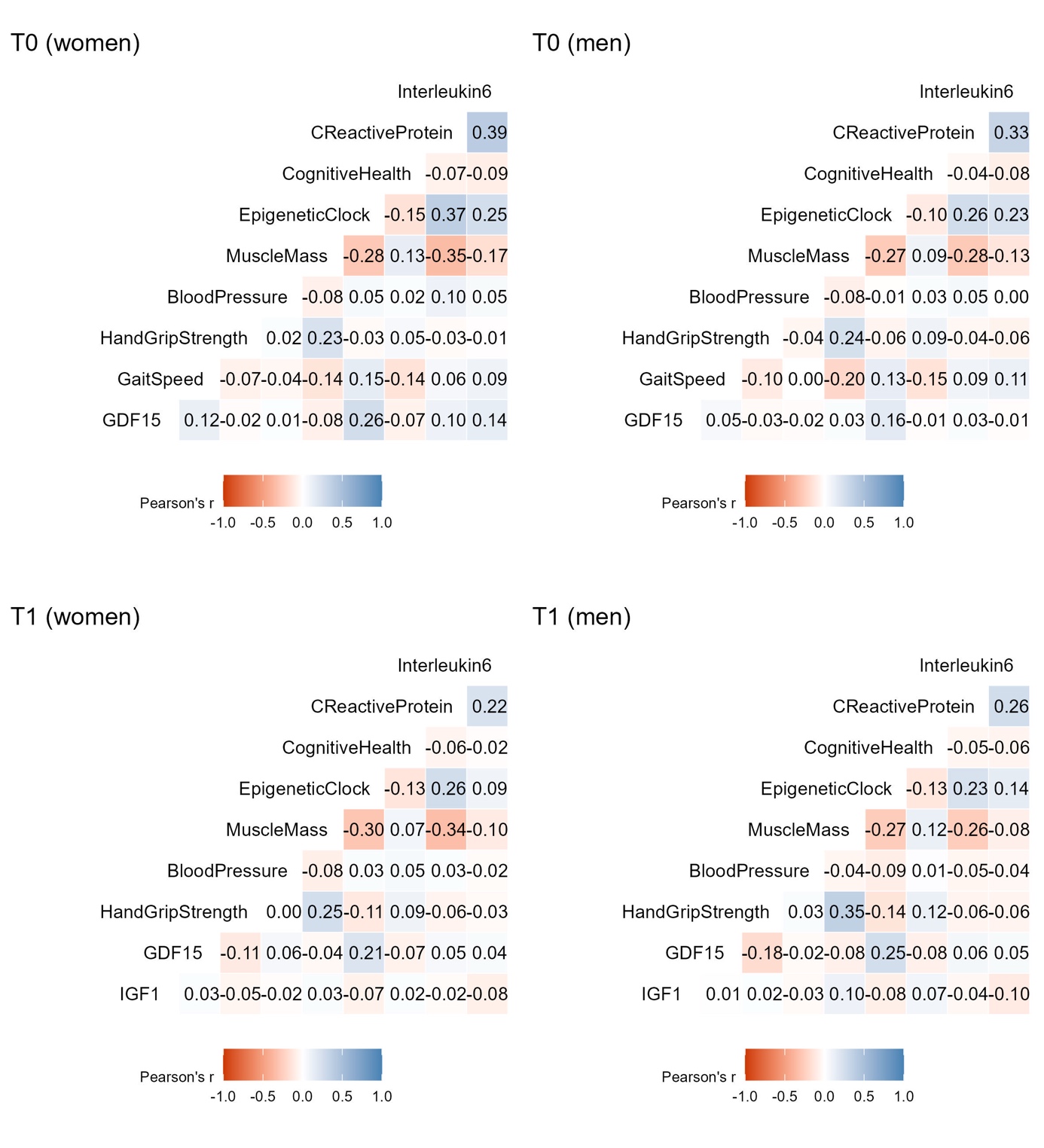


Supplementary Figure 1: Correlation plots (Pearson’s r) of continuously scaled biomarkers in sex-stratified subgroups at baseline (T0) and follow-up (T1) using the first imputed dataset.


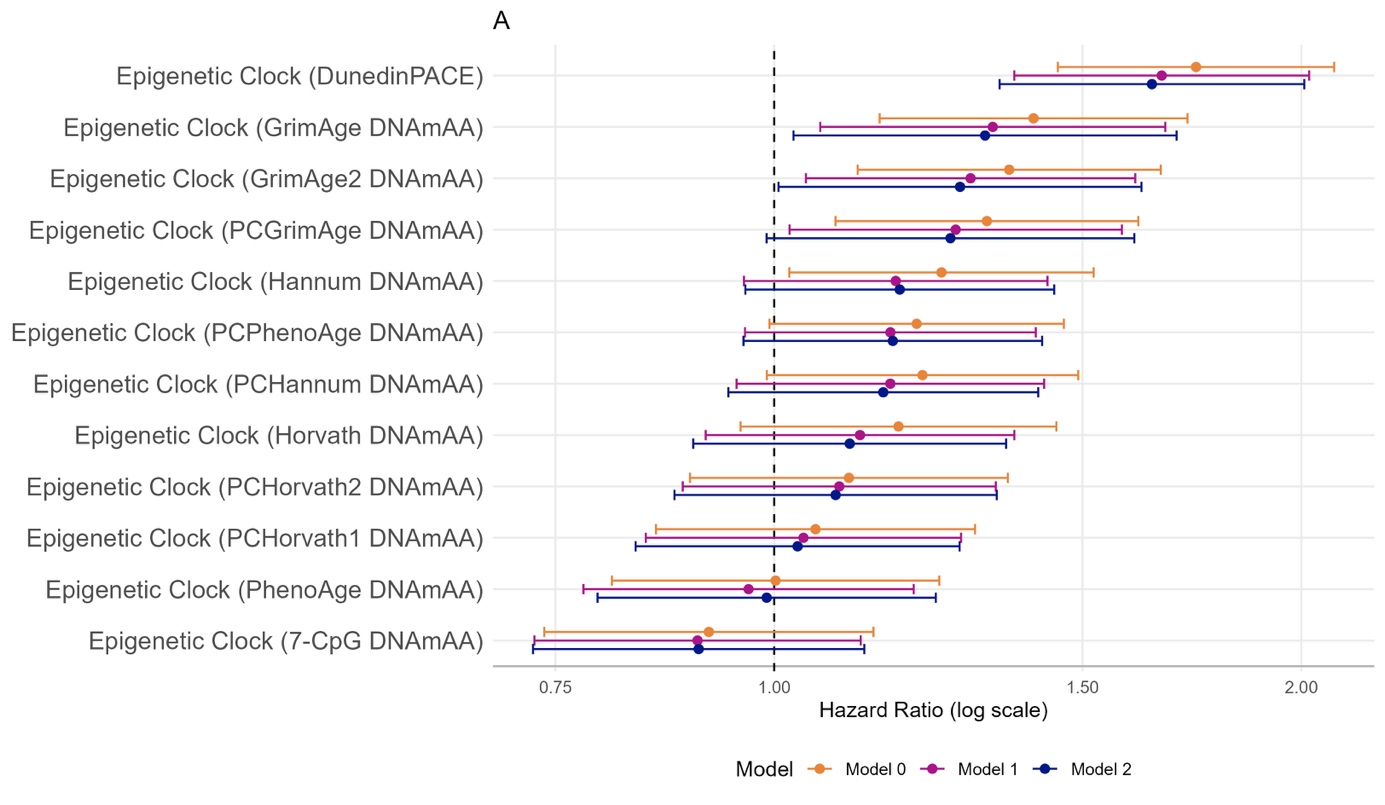


Supplementary Figure 2: Forest plot of results from Cox proportional hazard regression analysis of mortality according to epigenetic age estimates measured at T1 (n=1,083 participants, n=90 deaths). Model 0: unadjusted; Model 1: age, sex; Model 2: Model 1 + alcohol, smoking, physical activity, genetic heredity (PC1-PC4).

References:

Abramowitz, M. K., C. B. Hall, A. Amodu, D. Sharma, L. Androga and M. Hawkins (2018). "Muscle mass, BMI, and mortality among adults in the United States: a population-based cohort study." PloS one **13**(4): e0194697.

Adjoian Mezzaca, T., L. V. Dodds, T. Rundek, A. Zeki Al Hazzouri, M. R. Caunca, J. Gomes-Osman, D. A. Loewenstein, N. Schneiderman and T. Elfassy (2022). "Associations Between Cognitive Functioning and Mortality in a Population-Based Sample of Older United States Adults: Differences by Sex and Education." Journal of Aging and Health **34**(6-8): 905-915 DOI: 10.1177/08982643221076690.

Ascencio, E. J., G. D. Cieza-Gómez, R. M. Carrillo-Larco and P. J. Ortiz (2022). "Timed up and go test predicts mortality in older adults in Peru: a population-based cohort study." BMC Geriatrics **22**(1): 61 DOI: 10.1186/s12877-022-02749-6.

Boekholdt, S. M., C. E. Hack, M. S. Sandhu, R. Luben, S. A. Bingham, N. J. Wareham, R. J. G. Peters, J. W. Jukema, N. E. Day, J. J. P. Kastelein and K.-T. Khaw (2006). "C-reactive protein levels and coronary artery disease incidence and mortality in apparently healthy men and women: The EPIC-Norfolk prospective population study 1993–2003." Atherosclerosis **187**(2): 415-422 DOI: <https://doi.org/10.1016/j.atherosclerosis.2005.09.023>.

Cao, C., W. T. Cade, S. Li, J. McMillan, C. Friedenreich and L. Yang (2021). "Association of Balance Function With All-Cause and Cause-Specific Mortality Among US Adults." JAMA Otolaryngology–Head & Neck Surgery **147**(5): 460-468 DOI: 10.1001/jamaoto.2021.0057.

Cesari, M., S. B. Kritchevsky, A. B. Newman, E. M. Simonsick, T. B. Harris, B. W. Penninx, J. S. Brach, F. A. Tylavsky, S. Satterfield and D. C. Bauer (2009). "Added value of physical performance measures in predicting adverse health‐related events: results from the Health, Aging and Body Composition Study." Journal of the American Geriatrics Society **57**(2): 251-259.

Chang, S.-F. and P.-L. Lin (2015). "Frail phenotype and mortality prediction: A systematic review and meta-analysis of prospective cohort studies." International Journal of Nursing Studies **52**(8): 1362-1374 DOI: <https://doi.org/10.1016/j.ijnurstu.2015.04.005>.

Charlson, M. E., P. Pompei, K. L. Ales and C. R. MacKenzie (1987). "A new method of classifying prognostic comorbidity in longitudinal studies: development and validation." J Chronic Dis **40**(5): 373-383 DOI: 10.1016/0021-9681(87)90171-8.

Chen, Z., E. Nilsson, B. Lindholm, O. Heimbürger, P. Barany, P. Stenvinkel, A. R. Qureshi and J. Chen (2023). "Low-Plasma Insulin-Like Growth Factor-1 Associates With Increased Mortality in Chronic Kidney Disease Patients With Reduced Muscle Strength." J Ren Nutr **33**(2): 298-306 DOI: 10.1053/j.jrn.2022.06.008.

Chua, K. Y., W. S. Lim, X. Lin, J. M. Yuan and W.-P. Koh (2020). "Handgrip Strength and Timed Up-and-Go (TUG) Test are Predictors of Short-Term Mortality among Elderly in a Population-Based Cohort in Singapore." The Journal of nutrition, health and aging **24**(4): 371-378 DOI: <https://doi.org/10.1007/s12603-020-1337-0>.

Cooper, R., D. Kuh, R. Hardy and M. R. Group (2010). "Objectively measured physical capability levels and mortality: systematic review and meta-analysis." BMJ **341**: c4467 DOI: 10.1136/bmj.c4467.

Daniels, L. B., P. Clopton, G. A. Laughlin, A. S. Maisel and E. Barrett-Connor (2011). "Growth-Differentiation Factor-15 Is a Robust, Independent Predictor of 11-Year Mortality Risk in Community-Dwelling Older Adults." Circulation **123**(19): 2101-2110 DOI: doi:10.1161/CIRCULATIONAHA.110.979740.

De Giorgi, A., A. M. Marra, M. Iacoviello, V. Triggiani, G. Rengo, F. Cacciatore, C. Maiello, G. Limongelli, D. Masarone, F. Perticone, P. P. Filardi, S. Paolillo, A. Mancini, M. Volterrani, O. Vriz, R. Castello, A. Passantino, M. Campo, P. A. Modesti, A. Salzano, R. D'Assante, M. Arcopinto, V. Raparelli, F. Fabbian, A. Sciacqua, A. Colao, T. Suzuki, E. Bossone and A. Cittadini (2022). "Insulin-like growth factor-1 (IGF-1) as predictor of cardiovascular mortality in heart failure patients: data from the T.O.S.CA. registry." Intern Emerg Med **17**(6): 1651-1660 DOI: 10.1007/s11739-022-02980-4.

de Santana, F. M., M. O. Premaor, N. Y. Tanigava and R. M. R. Pereira (2021). "Low muscle mass in older adults and mortality: A systematic review and meta-analysis." Experimental Gerontology **152**: 111461 DOI: <https://doi.org/10.1016/j.exger.2021.111461>.

Föhr, T., K. Waller, A. Viljanen, T. Rantanen, J. Kaprio, M. Ollikainen and E. Sillanpää (2023). "Mortality Associations With DNA Methylation-Based Biological Aging and Physical Functioning Measures Across a 20-Year Follow-up Period." The Journals of Gerontology: Series A **78**(8): 1489-1496 DOI: 10.1093/gerona/glad026.

Fried, L. P., C. M. Tangen, J. Walston, A. B. Newman, C. Hirsch, J. Gottdiener, T. Seeman, R. Tracy, W. J. Kop and G. Burke (2001). "Frailty in older adults: evidence for a phenotype." The Journals of Gerontology Series A: Biological Sciences and Medical Sciences **56**(3): M146-M157 DOI: 10.1093/gerona/56.3.M146.

García-Hermoso, A., I. Cavero-Redondo, R. Ramírez-Vélez, J. R. Ruiz, F. B. Ortega, D.-C. Lee and V. Martínez-Vizcaíno (2018). "Muscular Strength as a Predictor of All-Cause Mortality in an Apparently Healthy Population: A Systematic Review and Meta-Analysis of Data From Approximately 2 Million Men and Women." Archives of Physical Medicine and Rehabilitation **99**(10): 2100-2113.e2105 DOI: <https://doi.org/10.1016/j.apmr.2018.01.008>.

Jochem, C., M. Leitzmann, K. Volaklis, D. Aune and B. Strasser (2019). "Association Between Muscular Strength and Mortality in Clinical Populations: A Systematic Review and Meta-Analysis." Journal of the American Medical Directors Association **20**(10): 1213-1223 DOI: <https://doi.org/10.1016/j.jamda.2019.05.015>.

König, M., M. Gollasch, I. Demuth and E. Steinhagen-Thiessen (2017). "Prevalence of Impaired Kidney Function in the German Elderly: Results from the Berlin Aging Study II (BASE-II)." Gerontology **63**(3): 201-209 DOI: 10.1159/000454831.

König, M., D. Spira, I. Demuth, E. Steinhagen-Thiessen and K. Norman (2018). "Polypharmacy as a risk factor for clinically relevant sarcopenia: results from the Berlin Aging Study II." The Journals of Gerontology: Series A **73**(1): 117-122.

Lee, J. K., R. Bettencourt, D. Brenner, T.-A. Le, E. Barrett-Connor and R. Loomba (2012). "Association between serum interleukin-6 concentrations and mortality in older adults: the Rancho Bernardo study." PloS one **7**(4): e34218.

Li, Y., X. Zhong, G. Cheng, C. Zhao, L. Zhang, Y. Hong, Q. Wan, R. He and Z. Wang (2017). "Hs-CRP and all-cause, cardiovascular, and cancer mortality risk: A meta-analysis." Atherosclerosis **259**: 75-82 DOI: <https://doi.org/10.1016/j.atherosclerosis.2017.02.003>.

Marioni, R. E., S. Shah, A. F. McRae, B. H. Chen, E. Colicino, S. E. Harris, J. Gibson, A. K. Henders, P. Redmond, S. R. Cox, A. Pattie, J. Corley, L. Murphy, N. G. Martin, G. W. Montgomery, A. P. Feinberg, M. D. Fallin, M. L. Multhaup, A. E. Jaffe, R. Joehanes, J. Schwartz, A. C. Just, K. L. Lunetta, J. M. Murabito, J. M. Starr, S. Horvath, A. A. Baccarelli, D. Levy, P. M. Visscher, N. R. Wray and I. J. Deary (2015). "DNA methylation age of blood predicts all-cause mortality in later life." Genome Biology **16**(1): 25 DOI: 10.1186/s13059-015-0584-6.

Meyer, A., B. Salewsky, D. Spira, E. Steinhagen-Thiessen, K. Norman and I. Demuth (2016). "Leukocyte telomere length is related to appendicular lean mass: cross-sectional data from the Berlin Aging Study II (BASE-II)." Am J Clin Nutr **103**(1): 178-183 DOI: 10.3945/ajcn.115.116806.

Pavlik, V. N., S. A. de Moraes, M. Szklo, D. S. Knopman, T. H. Mosley Jr. and D. J. Hyman (2003). "Relation between Cognitive Function and Mortality in Middle-aged Adults: The Atherosclerosis Risk in Communities Study." American Journal of Epidemiology **157**(4): 327-334 DOI: 10.1093/aje/kwf209.

Puzianowska-Kuźnicka, M., M. Owczarz, K. Wieczorowska-Tobis, P. Nadrowski, J. Chudek, P. Slusarczyk, A. Skalska, M. Jonas, E. Franek and M. Mossakowska (2016). "Interleukin-6 and C-reactive protein, successful aging, and mortality: the PolSenior study." Immunity & Ageing **13**(1): 21 DOI: 10.1186/s12979-016-0076-x.

Rahmani, J., A. Montesanto, E. Giovannucci, H. Zand, M. Barati, J. J. Kopchick, M. G. Mirisola, V. Lagani, H. Bawadi and R. Vardavas (2022). "Association between IGF‐1 levels ranges and all‐cause mortality: A meta‐analysis." Aging Cell **21**(2): e13540.

Rolland, Y., V. Lauwers-Cances, M. Cesari, B. Vellas, M. Pahor and H. Grandjean (2006). "Physical Performance Measures as Predictors of Mortality in a Cohort of Community-dwelling Older French Women." European Journal of Epidemiology **21**(2): 113-122 DOI: 10.1007/s10654-005-5458-x.

Rosano, C., A. B. Newman, R. Katz, C. H. Hirsch and L. H. Kuller (2008). "Association between lower digit symbol substitution test score and slower gait and greater risk of mortality and of developing incident disability in well‐functioning older adults." Journal of the American Geriatrics Society **56**(9): 1618-1625.

Satish, S., D. H. Freeman Jr, L. Ray and J. S. Goodwin (2001). "The relationship between blood pressure and mortality in the oldest old." Journal of the American Geriatrics Society **49**(4): 367-374.

Spira, D., N. Buchmann, J. Nikolov, I. Demuth, E. Steinhagen-Thiessen, R. Eckardt and K. Norman (2015). "Association of Low Lean Mass With Frailty and Physical Performance: A Comparison Between Two Operational Definitions of Sarcopenia-Data From the Berlin Aging Study II (BASE-II)." Spira, Dominik, et al. "Association of low lean mass with frailty and physical performance: a comparison between two operational definitions of sarcopenia—data from the Berlin Aging Study II (BASE-II)." Journals of Gerontology Series A: Biomedical Sciences and Medical Sciences **70**(6): 779-784 DOI: 10.1093/gerona/glu246.

Studenski, S., S. Perera, K. Patel, C. Rosano, K. Faulkner, M. Inzitari, J. Brach, J. Chandler, P. Cawthon, E. B. Connor, M. Nevitt, M. Visser, S. Kritchevsky, S. Badinelli, T. Harris, A. B. Newman, J. Cauley, L. Ferrucci and J. Guralnik (2011). "Gait Speed and Survival in Older Adults." JAMA **305**(1): 50-58 DOI: 10.1001/jama.2010.1923.

Todd, O. M., C. Wilkinson, M. Hale, N. L. Wong, M. Hall, J. P. Sheppard, R. J. McManus, K. Rockwood, J. Young, C. P. Gale and A. Clegg (2019). "Is the association between blood pressure and mortality in older adults different with frailty? A systematic review and meta-analysis." Age and Ageing **48**(5): 627-635 DOI: 10.1093/ageing/afz072.

Toepfer, S., J. Bolbrinker, M. König, E. Steinhagen-Thiessen, R. Kreutz and I. Demuth (2019). "Potentially inappropriate medication in older participants of the Berlin Aging Study II (BASE-II)–sex differences and associations with morbidity and medication use." PLoS One **14**(12): e0226511.

Vermeiren, S., R. Vella-Azzopardi, D. Beckwée, A.-K. Habbig, A. Scafoglieri, B. Jansen, I. Bautmans, I. Bautmans, D. Verté, I. Beyer, M. Petrovic, L. De Donder, T. Kardol, G. Rossi, P. Clarys, A. Scafoglieri, E. Cattrysse, P. de Hert and B. Jansen (2016). "Frailty and the Prediction of Negative Health Outcomes: A Meta-Analysis." Journal of the American Medical Directors Association **17**(12): 1163.e1161-1163.e1117 DOI: <https://doi.org/10.1016/j.jamda.2016.09.010>.
